# Editorial: Microbial Systems as Paradigms of Successful and Sustainable Interactions

**DOI:** 10.3389/fmicb.2021.785106

**Published:** 2021-11-24

**Authors:** Nathalie Connil, Anna Luganini, Enrica Pessione

**Affiliations:** ^1^Laboratory of Microbiology Signals and Microenvironment (LMSM EA 4312), University of Rouen, Evreux, France; ^2^Laboratory of Microbiology and Virology, Department of Life Sciences and Systems Biology, University of Torino, Turin, Italy; ^3^Laboratory of Microbial Applied Biochemistry and Proteomics, Department of Life Sciences and Systems Biology, University of Torino, Turin, Italy

**Keywords:** microbial ecosystems, biofilms, resilience, evolution, cooperation, cross-feeding, molecular signaling

The idea at the basis of the present Research Topic was to explore how evolution selected microbial behaviors to ensure the generation of more and more complex and sustainable lifestyles that help microbial systems in coping with scarcity and rapidly changing environments. The keywords were communication, interaction, cooperation, adaptation, energy earning and complexity. The rich web of interactions that characterize microbial life can represent a paradigm of the concept “the whole is more than the sum of its parts” and the interactive lifestyle is the best one ensuring survival and evolution. It is well-assessed that, besides vertical inheritance, signal exchange, horizontal gene transfer and epigenetic events account for the fast evolution and plasticity observed (Bapteste, 2014). Understanding these interspecies and inter-kingdom relationships, as well as those with the external environment unravels the underlying rule of these interactions: sense the enemy/constraint, cope and adjust to acquire novel lifestyle opportunities. Thus, observing microbial systems can inspire us offering old solutions to new emerging problems.

Bacteria have a long evolutionary history both as planktonic cells and as parts of microbial consortia such as biofilms. They can live in the external environment in abiotic habitats but they can also colonize different animal and vegetal districts to establish a symbiotic way of living with higher organisms. The evolutionary pressure has selected bacterial behaviors intended to facilitate both reciprocal and inter-kingdom interactions. All these relationships have the peculiarity to be cooperatively successful and sustainable (long-lasting).

In the present Research Topic, different aspects of bacteria lifestyles are explored. First, their reciprocal interactions (which include cooperation but also conflicts) that are made-up by sustainable behaviors like earning energy and resources, optimize rather than maximize, anticipate, sharing, and recycling. In this context direct connections (biofilm) but also soluble and vesicle-embedded signals are used to communicate (Pessione). A paradigmatic example are the interactions and the exchange of signals in a multispecies biofilm characterized by high metabolic heterogeneity. Generally, cross-feeding, ensuring removal of toxic metabolic products, allows enhanced growth rates, however, these biofilm-living communities can affect also the interaction with the human host (Joshi et al.). In addition, metabolite exchange (thiamine) occurring in solvent-degrading bacterial communities is reported as an example of syntrophy among species (Huang et al.). Secondarily, bacterial plasticity and ability to interact with different animal models such as mammals and crabs is analyzed. An interesting example is the study of lemur's gut microbiome. Correa et al. reported that bacterial alpha and beta diversity are affected by family group and sex. Furthermore, the contribution of gut bacteria to lemurs' evolution, by processing diet-acquired strategic molecules or ions that the animal up-takes directly from the ground is reported, suggesting an ecological value of soil as a nutrient supply. As far as the human host is concerned, symbiont bacteria can establish inter-kingdom communication by affecting brain signaling (bottom up control of the gut-brain axis) but also by responding to top-down signals, demonstrating that host and microbiota share the same molecular language, in agreement with the concept of economizing molecules by using multitasking signals (Boukerb et al.). Another crucial function of symbiont bacteria is to allow to crabs the transition from water world to terrestrial life permitting the utilization of novel food sources. This is achieved by means of cellulolytic symbiont bacteria ensuring the availability of free mono- and disaccharides promoting a carbohydrate based diet instead of protein-based meals for the host crab (Cannicci et al.). Interestingly, crabs living in different aquatic ecological niches host a different commensal microbiota displaying diversity especially in fatty acid profiles (Su et al.).

Apart from bacteria, other microorganisms such as yeasts can set up a successful lifestyle by interacting with insects: the well-established symbiosis between *Saccharomyces cerevisiae* and social wasps provides advantages to both partners favoring yeast mating (a not frequent event in natural environments) and modulating the immune system of insect host (Meriggi et al.).

An interesting example of community fitness is the one of lichens. They can colonize extreme environments (boreal forest, arctic tundra, lava fields and tide-flooded areas) and they are good bio-indicators for monitoring pollution by heavy metals or sulfur dioxide. However, some lichens prove to be resistant to sulfur dioxide because of their surface hydrophobicity (Hauck et al., 2008) and to metals due to oxalate production (Purvis, 2014). In this Research Topic, two articles dealing with lichens are included. The former (Grimm et al.), underlines the importance of all “omics” approaches and molecular imaging in deciphering the complex structure and physiology of these interacting communities. Metaproteome analyses confirm the partition of functions in lichen partnerships also highlighting the (still poorly explored) diversity of the lichen microbiota and the additional roles of commensal bacteria and viruses in the lichen ecosystem, and describes interesting aspects of the bacterial microbiota in supporting nutrient uptake, adaptability, stress tolerance and in coping with exogenous pathogenic bacteria. These lichen social systems, whose composition differs in different geographical sites, prove to be excellent inter-kingdom aggregations able to adapt to climate changes and displaying resilience to external disturbances underlining the concept “Unity is strength” (Grimm et al.). The second article (Nazem-Bokaee et al.) mainly explores the lichen symbiosis by network modeling using the tools of systems biology for analyzing the metabolic interplays and the underlying molecular mechanisms of lichen signaling pathways. This holistic approach, going beyond the genomic information, highlights the importance of deciphering the flux distribution in lichen metabolic pathways and the co-dependence between symbionts.

Finally, the role of viruses, in establishing successful interactions supporting human fitness and evolution is explored in two reviews of this Topic Issue. Endogenization of retroviruses is crucial in human evolution, for instance in the placenta formation but also when retroviruses exert a neuroprotective effect on the human brain. The article by Luganini and Gribaudo provides amazing examples of cooperation essential for survival that strongly support the idea that each infection could be an opportunity of evolution, mediated by mobile genetic elements. This is also the main thread that emerges when considering the complex and multilevel interaction between phages, bacteria and the animal host: like a Russian Doll model, bacteria can talk with their inner (phage) and outer (human) hosts. Phage infection can offer to bacteria weapons to cope with competing microbial species and with the host immune system. Phage populations in the human gut also help the host to select beneficial symbiont bacteria. In addition, bacteria can communicate with their animal host by means of multitasking moonlighting proteins and by using post-translational modifications that affect the host perception of the microbial commensal and that can alter host pathways and gene expression as well (Pessione).

Taken together all these data suggest that every interaction among microbes and between microbes and higher organisms can be an opportunity of evolution, generating a higher degree of complexity. Moreover, evolution seems to have selected all those behaviors that support collective resilience, adaptation and other winning strategies to cope with a crowded environment and to counteract fast changing conditions like those observable in this period of climate change and global warming. Nevertheless, it can be highlighted that this Research Topic is an incomplete work: other aspects for instance the symbioses in the vegetal world or the interactions occurring in the aquatic ecosystems can add other bricks to this fascinating world.

## Author Contributions

EP wrote the first draft of the manuscript. AL, NC, and EP contributed to manuscript revision, read, and approved the submitted version. All authors contributed to the article and approved the submitted version.

## Funding

This work was supported by grants from Ricerca Locale from the University of Turin (PESE_RILO 20_01 to EP and LUGA_RILO_21_01 to AL).

## Conflict of Interest

The authors declare that the research was conducted in the absence of any commercial or financial relationships that could be construed as a potential conflict of interest.

## Publisher's Note

All claims expressed in this article are solely those of the authors and do not necessarily represent those of their affiliated organizations, or those of the publisher, the editors and the reviewers. Any product that may be evaluated in this article, or claim that may be made by its manufacturer, is not guaranteed or endorsed by the publisher.
